# Chronic hyperglycaemia increases the vulnerability of the hippocampus to oxidative damage induced during post-hypoglycaemic hyperglycaemia in a mouse model of chemically induced type 1 diabetes

**DOI:** 10.1007/s00125-023-05907-6

**Published:** 2023-04-04

**Authors:** Alison D. McNeilly, Jennifer R. Gallagher, Mark L. Evans, Bastiaan E. de Galan, Ulrik Pedersen-Bjergaard, Bernard Thorens, Albena T. Dinkova-Kostova, Jeffrey-T. Huang, Michael L. J. Ashford, Rory J. McCrimmon, on behalf of the Hypo-RESOLVE Consortium

**Affiliations:** 1 Division of Systems Medicine, School of Medicine, Ninewells Hospital and Medical School, Dundee, UK; 2 Wellcome-MRC Institute of Metabolic Science, University of Cambridge, Cambridge, UK; 3 Radboud University Medical Center, Nijmegen, the Netherlands; 4 Department of Internal Medicine, Maastricht University Medical Center, Maastricht, the Netherlands; 5 CARIM School for Cardiovascular Diseases, Maastricht University, Maastricht, the Netherlands; 6 Nordsjællands Hospital Hillerød, University of Copenhagen, Hillerød, Denmark; 7 Faculty of Biology and Medicine, University of Lausanne, Lausanne, Switzerland; 8 Division of Cancer Research, School of Medicine, Ninewells Hospital and Medical School, Dundee, UK; 9 Biomarker and Drug Analysis Core Facility, School of Medicine, Ninewells Hospital and Medical School, Dundee, UK

**Keywords:** Glycaemic variability, Hippocampus, Hyperinsulinaemic glucose clamp, Hypoglycaemia, Mouse, *Nfe2l2*, *Nrf2*, Oxidative stress, Proteotoxic stress, Type 1 diabetes

## Abstract

**Aims/hypothesis:**

Chronic hyperglycaemia and recurrent hypoglycaemia are independently associated with accelerated cognitive decline in type 1 diabetes. Recurrent hypoglycaemia in rodent models of chemically induced (streptozotocin [STZ]) diabetes leads to cognitive impairment in memory-related tasks associated with hippocampal oxidative damage. This study examined the hypothesis that post-hypoglycaemic hyperglycaemia in STZ-diabetes exacerbates hippocampal oxidative stress and explored potential contributory mechanisms.

**Methods:**

The hyperinsulinaemic glucose clamp technique was used to induce equivalent hypoglycaemia and to control post-hypoglycaemic glucose levels in mice with and without STZ-diabetes and *Nrf2*^−/−^ mice (lacking *Nrf2* [also known as *Nfe2l2*]). Subsequently, quantitative proteomics based on stable isotope labelling by amino acids in cell culture and biochemical approaches were used to assess oxidative damage and explore contributory pathways.

**Results:**

Evidence of hippocampal oxidative damage was most marked in mice with STZ-diabetes exposed to post-hypoglycaemic hyperglycaemia; these mice also showed induction of *Nrf2* and the *Nrf2* transcriptional targets *Sod2* and *Hmox-1*. In this group, hypoglycaemia induced a significant upregulation of proteins involved in alternative fuel provision, reductive biosynthesis and degradation of damaged proteins, and a significant downregulation of proteins mediating the stress response. Key differences emerged between mice with and without STZ-diabetes following recovery from hypoglycaemia in proteins mediating the stress response and reductive biosynthesis.

**Conclusions/interpretation:**

There is a disruption of the cellular response to a hypoglycaemic challenge in mice with STZ-induced diabetes that is not seen in wild-type non-diabetic animals. The chronic hyperglycaemia of diabetes and post-hypoglycaemic hyperglycaemia act synergistically to induce oxidative stress and damage in the hippocampus, possibly leading to irreversible damage/modification to proteins or synapses between cells. In conclusion, recurrent hypoglycaemia in sub-optimally controlled diabetes may contribute, at least in part, to accelerated cognitive decline through amplifying oxidative damage in key brain regions, such as the hippocampus.

**Data availability:**

The datasets generated during and/or analysed during the current study are available in ProteomeXchange, accession no. 1-20220824-173727 (www.proteomexchange.org). Additional datasets generated during and/or analysed during the present study are available from the corresponding author upon reasonable request.

**Graphical abstract:**

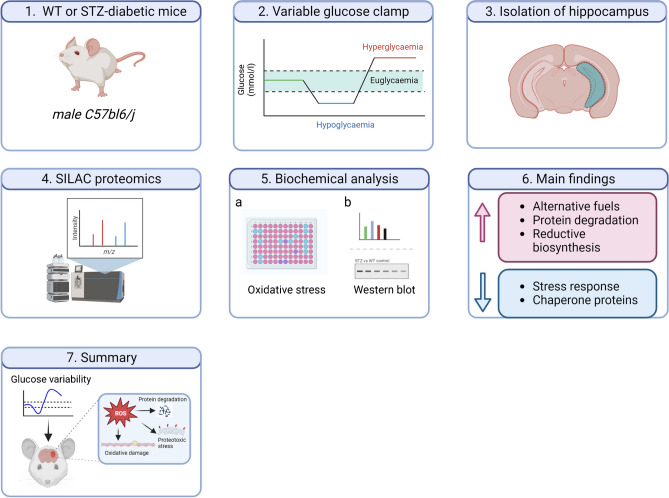

**Supplementary Information:**

The online version contains peer-reviewed but unedited supplementary material available at 10.1007/s00125-023-05907-6.

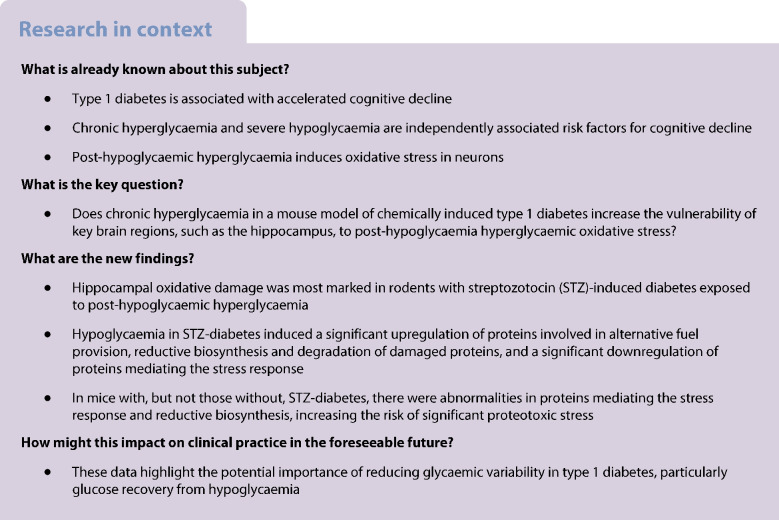

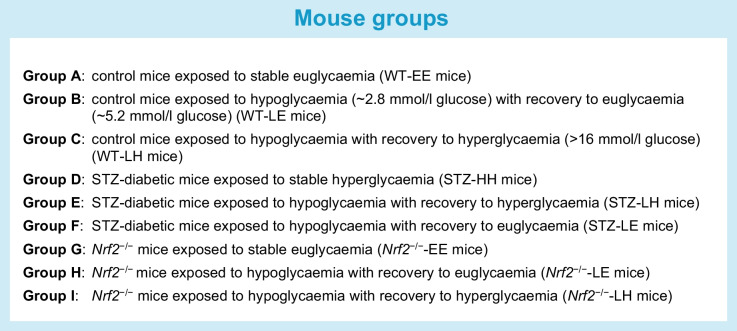





## Introduction

Short-duration longitudinal studies in young adults with type 1 diabetes compared with matched adults without diabetes have reported small but significant increases in the rate of cognitive decline associated with proliferative retinopathy and systolic hypertension [1, 2]. More recently, the 32-year follow-up of participants enrolled in the DCCT/Epidemiology of Diabetes Interventions and Complications (EDIC) study reported that higher HbA_1c_ levels over time and elevated systolic BP were associated with a greater rate of cognitive decline collectively equivalent to 9.4 years accelerated brain ageing [3]. Within the EDIC cohort, an fMRI substudy of middle-aged and older adults found brain volume loss and increased vascular injury compared with control individuals without diabetes [4]. Severe hypoglycaemia was also reported to be independently associated with cognitive decline in the EDIC cohort [3], a finding consistent with observational [5–7] and short-duration longitudinal [8] studies.

A reliance on glucose as a fuel, and limited capacity to store fuel, makes the brain especially vulnerable to hypoglycaemia [9]. Certainly, profound hypoglycaemia (to a degree that induces an isoelectric EEG) results in neuronal death in areas of the brain such as the hippocampus [10]. The hippocampus has been extensively researched for its role in memory function, processing speed, and intelligence [11]. Cognitive ageing is associated with loss of hippocampal volume [12]. In a recent report from our laboratory, we showed that rodents with chemically induced type 1 diabetes who had been exposed to recurrent hypoglycaemia demonstrated greater defects in memory function than rodents with type 1 diabetes who had not experienced recurrent hypoglycaemia. This was associated with evidence of lipid peroxidation and protein carbonylation in the hippocampus, markers of oxidative damage [13].

Although reactive oxygen species (ROS) play an integral part in the normal signalling response within many cell types, including neurons, large and frequent disturbances in glucose homeostasis cause excessive ROS production resulting in oxidative stress [14, 15]. Chronic hyperglycaemia [16], severe hypoglycaemia [17] and post-hypoglycaemic glucose recovery all stimulate ROS production. Notably, ROS production post-hypoglycaemia correlates directly with the degree of glucose increase during recovery from hypoglycaemia [18]. Chronic hyperglycaemia also impairs antioxidant defence mechanisms [19, 20]. This led us to hypothesise that marked glycaemic variability may lead to excessive ROS production and irreversible oxidative damage to cells within the brain [13]. What is not clear from these studies is the relative contribution of each to oxidative damage and the key pathways that may underlie this. In the present study, we address this question directly using the hyperinsulinaemic glucose clamp technique combined with the measurement of ROS-induced protein modifications (protein carbonylation and lipid peroxidation) and stable isotope labelling by amino acids in cell culture (SILAC) proteomic analysis of the hippocampus in a variety of mouse models.

## Methods

### Experimental animals

Male C576BL/6J mice (20–25 g; Charles River, UK) were used. The generation (mice were backcrossed over six generations onto a C57BL/6J background) and genotyping of *Nrf2*^−/−^ mice lacking *Nrf2* (also known as *Nfe2l2*), kindly provided by K. Itoh and M. Yamamoto (Centre for Tsukuba Advanced Research Alliance and Institute of Basic Medical Sciences, University of Tsukuba, Tsukuba, Japan), were performed as described previously [21]. Mice were housed four per cage with food and water available ad libitum, on a 12 h light–dark schedule. All animal procedures were approved by the University of Dundee Ethical Review Process and performed according to UK Home Office regulations and the ARRIVE 2.0 guidelines (under the auspices of Project License PIL PE82c1898).

### Induction of diabetes

C576BL/6J mice were randomly assigned to receive streptozotocin (STZ; 150 mg/kg i.p.) to chemically induce STZ-diabetes or control (Hanks’ Buffered Salt Solution buffer; Gibco, UK; i.p.). At 72 h and 7 days post-injection, blood glucose was measured from tail-vein samples using a hand-held glucose monitor (Accuread, Roche, UK); blood glucose ≥16.0 mmol/l was regarded as diabetic. Any mouse that failed to reach this criterion was given a second injection of STZ, and blood glucose was re-tested. To maintain body weight and health, Linbit insulin implants (LinShin, Canada; at half of the recommended dose [~0.05 U/kg per day]) were inserted subcutaneously under isoflurane anaesthetic as described [13]. Control mice were also anaesthetised.

### Vascular surgery and glycaemic clamping

After 4 weeks of stable hyperglycaemia (STZ-diabetes) or euglycaemia (wild-type [WT] control and *Nrf2*^−/−^mice), the mice underwent surgery for the insertion of vascular catheters as described previously [22]. Mice were allowed to recover for 5 days (or until they reached pre-surgery weight).

### Infusion protocol

**Fig. 1:**
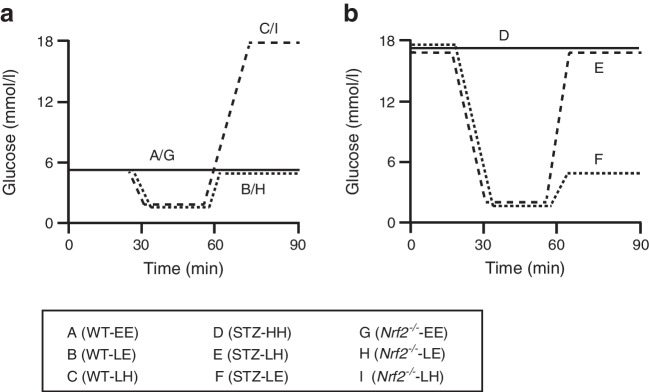
Experimental design of mouse glycaemic clamps. (**a**) Control (A, B, C) and *Nrf2*^−/−^ (G, H, I) mice were exposed to stable euglycaemia (WT- or *Nrf2*^−/−^-EE), hypoglycaemia (~ 2.8 mmol/l) with recovery to euglycaemia (~5.2 mmol/l) (WT- or *Nrf2*^−/−^-LE), or hypoglycaemia with recovery to hyperglycaemia (>16 mmol/l) (WT- or *Nrf2*^−/−^-LH). (**b**) STZ-diabetic (D, E, F) mice were exposed to stable hyperglycaemia (STZ-HH), hypoglycaemia with recovery to hyperglycaemia (STZ-LH), or hypoglycaemia with recovery to euglycaemia (STZ-LE)

As previously described, a 2 h 4 mU kg^−1^ min^−1^ infusion of human short-acting insulin (Actrapid, Novo Nordisk, UK) was initiated in mice fasted for 5 h [23]. Mice were then allocated into groups (see Fig. 1 and the Text box detailing mouse groups). Target glucose levels (5.2 mmol/l [euglycaemia], 2.8 mmol/l [hypoglycaemia] and >16 mmol/l [hyperglycaemia]) were achieved and maintained for at least 30 min using a variable 50% glucose infusion based on frequent plasma glucose determinations. Additional blood samples to measure counterregulatory hormones were taken at the end of the second step of the clamp. At the end of the clamp, the mice were given food and water ad libitum and allowed to recover to their endogenous glucose levels (i.e., hyperglycaemia for STZ-diabetes mice and euglycaemia for WT control and *Nrf2*^−/−^ mice).

### Hormone analysis

Plasma glucagon and adrenaline levels were measured using commercially available ELISA kits (adrenaline, DEE5100R, Demeditec, Germany; glucagon, 10-1281-01, Mercodia, Sweden).

### Biochemical analysis

Sixteen hours after the clamp, mice were killed humanely, and the brain (hippocampus) was dissected and flash-frozen in liquid nitrogen for subsequent biochemical analysis.

### Sample preparation and SILAC

Hippocampal samples (single lobe from each mouse) from groups A (WT-EE) and E (STZ-LH) were analysed using SILAC-based proteomic analysis [24]. Samples were randomised, and the analysts were blinded during data acquisition. Samples were homogenised, and the protein extracted in sodium dodecyl sulfate (SDS, 4% wt/vol.) dithiothreitol (DTT, 0.1% wt/vol.), 100 mmol/l Tris-HCl (pH 7.6). After centrifugation, protein lysates from each experimental sample were spiked with an equivalent amount of SILAC protein lysate. After heating to 60°C for 30 min, the samples were alkylated by adding an equal volume of 150 mmol/l iodoacetamide (in 100 mmol/l Tris-HCl buffer [pH 7.6]). The protein was precipitated using the MeOH–chloroform method [25], and the protein concentration was measured using Protein 660 nm reagent (Pierce, UK). Samples were reduced/alkylated, digested with LysC (Pierce, 1:100), and then fractionated using a strong anion exchanger [26].

### LC-MS/MS and data processing

The top 6 ms/ms programs (collision-induced dissociation [CID] or pulse-Q dissociation [PQD]) on LTQ-Orbitrap (Thermo Scientific, Germany) peptide identification and protein quantification were assessed using Maxquant (Ver 1.5.0.30; www.maxquant.org) or PEAKS 7.0 (Bioinformatics Solution Inc, Canada; database =Uniprot mouse 2017-02-29). Quantification is based on the methods described [24]. Missing data points were replaced (with 0) only for principal component analysis (PCA). A false detection rate (FDR) was set to 1% at the identified peptide spectrum match level. N-terminal acetylation, cysteine carbamidomethylation, and phosphorylation at S/T/Y were the only permitted post-translational modifications. Normalisation was performed using *z*-score normalisation in Perseus (Version 1.5.4.0, www.maxquant.org).

### Western blot analysis

The second hippocampal lobe from all groups was powdered on liquid nitrogen using a pestle and mortar. A portion of the powder was homogenised in lysis buffer containing protease inhibitors and prepared for western blotting or proteomics analysis as described [27]. The remaining powder was frozen at −80°C for subsequent biochemical analysis. Membranes were probed for the following target proteins identified from SILAC (all from Cell Signalling Technology, UK): heat shock protein 90-β (HSP90B); proteasome subunit α type-2 (PSMA2); proteasome subunit α type-3 (PSMA3); proteasome subunit β type-7 (PSMB7); 6-phosphogluconate dehydrogenase (6PGD); long-chain acyl Co-A dehydrogenase (ACADL); and HSP90 co-chaperone (CDC37). All blots were normalised to the housekeeping protein GAPDH.

### Lipid peroxidation

Malondialdehyde concentration was determined in all hippocampal samples (A–I) by the thiobarbituric acid-reactive substances assay [28] using a 96-well plate format. The amount of malondialdehyde was determined spectrophotometrically at 532 nm, and concentrations were determined by standard curve. All samples were assayed in duplicate.

### Protein carbonylation

The level of carbonylated protein within the hippocampus of all groups (A–I) was measured by ELISA (Caymen Chemicals, US). Protein carbonyl concentration was calculated using the following equation: protein carbonyl (nmol/ml) = CA/(× 0.011 [mmol/l]^−1^) (500 ml/200 ml), where CA is the corrected absorbance (mean absorbance of controls – mean absorbance of samples).

### RNA extraction and PCR

Total RNA was extracted from hippocampal tissue from all groups (A–I) using TRIzol reagent (Invitrogen, UK). Reverse transcription was performed with 1 ng RNA using SuperScript III First-Strand Synthesis System for RT (Invitrogen). Real-time PCR was performed using TaqMan gene expression assays for the following genes: *Nrf2* (encoding for nuclear factor erythroid 2-related factor 2 [NRF2]); *Nqo1* (encoding for NAD(P)H: quinone oxidoreductase 1); *Hmox-*1 (encoding for haem oxygenase 1); and *Sod2* (encoding for superoxide dismutase 2)*.* All samples were performed in triplicate and normalised to the housekeeping genes *Actb* and *Ppia*. Values are expressed as a fold-change relative to group A (WT-EE) for STZ-diabetic mice and group G (*Nrf2*^*−/−*^-EE) for *Nrf2*^−/−^ mice.

### Statistical analysis

Data were analysed using SPSS version 18 (IBM, UK). One-way ANOVA was used to compare clamp groups within each genotype (groups A–F for control [WT] and STZ-diabetes mice; groups G–I for *Nrf2*^−/−^ mice). Post hoc analysis was performed using Tukey’s multiple comparisons test. For data that were not normally distributed, Kruskal–Wallis, followed by Dunn’s multiple comparisons test, was performed. Data are expressed as mean values ± SEM. Statistical significance was set at *p*<0.05.

## Results

### Hyperinsulinaemic clamp studies on control and STZ-diabetic mice

Stable hypoglycaemic (groups B, C, E and F) and hyperglycaemic plateaus (groups C, D, E and F) were achieved during the clamp procedures (Table 1; *p*<0.05 for each group vs WT control [group A]). In groups B and C, hypoglycaemia from a euglycaemic baseline resulted in significantly elevated glucagon and adrenaline plasma levels compared with group A. In contrast, in the STZ-diabetic mice (groups E and F), consistent with human type 1 diabetes, the glucagon response to a hypoglycaemic challenge was impaired (Table 1) [29] and the adrenaline response was severely blunted. The hormonal counterregulatory response to hypoglycaemia in *Nrf2*^−/−^ mice was comparable with that in C57Bl6/J control mice (Table 1).

**Table 1:** Mean plasma glucose levels during each phase of the hyperinsulinaemic glucose clamps along with counterregulatory hormone levels (glucagon and adrenaline) measured at the end of the eu/hypoglycaemia period

Group	Mean glucose (mmol/l)			Glucagon (ng/l)	Adrenaline (pg/ml)
WT					
A	WT	E^a^	E	E^a^	E^a^
	5.6±0.4	5.4±0.2	5.7±0.3	35±3	175±43.7
B	WT	L^a^	E	L^a^	L^a^
	5.4±0.3	2.7±0.3*	5.6±0.4	143±10**	802±81.9**
C	WT	L^a^	H	L^a^	L^a^
	5.8±0.5	2.6±0.1*	20.8±0.4**	135±8**	770±65.5**
STZ-diabetes					
D	STZ	H^a^	H	H^a^	H^a^
	19.3±1.0**	18.3±2.2**	18.8±2.3**	22±6^¶¶^	267±49.1^¶¶^
E	STZ	L^a^	H	L^a^	L^a^
	22.1±2.9**	3.3±0.6*	21.3±1.9**	48±12^¶¶^	333±92.8^¶¶^
F	STZ	L^a^	E	L^a^	L^a^
	20.9±1.8**	2.9±0.7*	6.1±2.4	43±13^¶¶^	355±81.9^¶¶^
*Nrf2*^−/−^					
G	*Nrf2*	E^a^	E	E^a^	E^a^
	6.3±0.3	6.3±0.4	6.3±0.3	42±6	251±38.2
H	*Nrf2*	L^a^	E	L^a^	L^a^
	6.2±0.2	2.5±0.1^†^	6.4±0.2	116±12^††^	704±54.6^††^
I	*Nrf2*	L^a^	H	L^a^	L^a^
	6.2±0.3	2.6±0.1^†^	17.1±0.5^††^	125±8^††^	753±81.9^††^

Results represent mean values ± SEM, *n*=10–12 per group

^a^Glucose level during which the hyperinsulinaemic clamp was maintained and glucagon and adrenaline measurements were made

*p<0.05, **p<0.01 vs group A; ^¶¶^*p*<0.01 vs both groups B and C; ^†^*p*<0.05, ^††^*p*<0.01 vs group G (one-way ANOVA followed by Tukey’s multiple comparisons test)

E, euglycaemia ~5.2 mmol/l; H, high, hyperglycaemia >16.0 mmol/l; L, low, hypoglycaemia ~2.8 mmol/l, Nrf2, *Nrf2*^−/−^

### Chronic hyperglycaemia acts synergistically with acute hypoglycaemia to induce NRF2 target genes

To examine the impact of hypoglycaemia on *Nrf2* and NRF2 target genes *Nqo1*, *Sod2* and *Hmox-1*, their expression levels were measured in the hippocampus of all control and STZ-diabetes groups (electronic supplementary material [ESM] Table 1). Transcript levels of *Nqo1* and *Sod2* were significantly elevated in STZ-diabetic mice following acute hypoglycaemia (STZ-LH vs WT-EE; *p*<0.05 for both genes), and the levels of *Sod2* were further increased (>fivefold) in chronic hyperglycaemia. In WT non-diabetic mice, *Sod2 and Hmox-1* transcript levels were significantly elevated by hypoglycaemia (WT-LE vs WT-EE; *p*<0.05). As anticipated, RNA levels of these NRF2 target genes were unaltered in *Nrf2*-knockout mice (ESM Table 2), demonstrating NRF2 dependence.

### Acute hypoglycaemia in STZ-diabetic mice but not in non-diabetic WT mice induces oxidative damage in the hippocampus

**Fig. 2:**
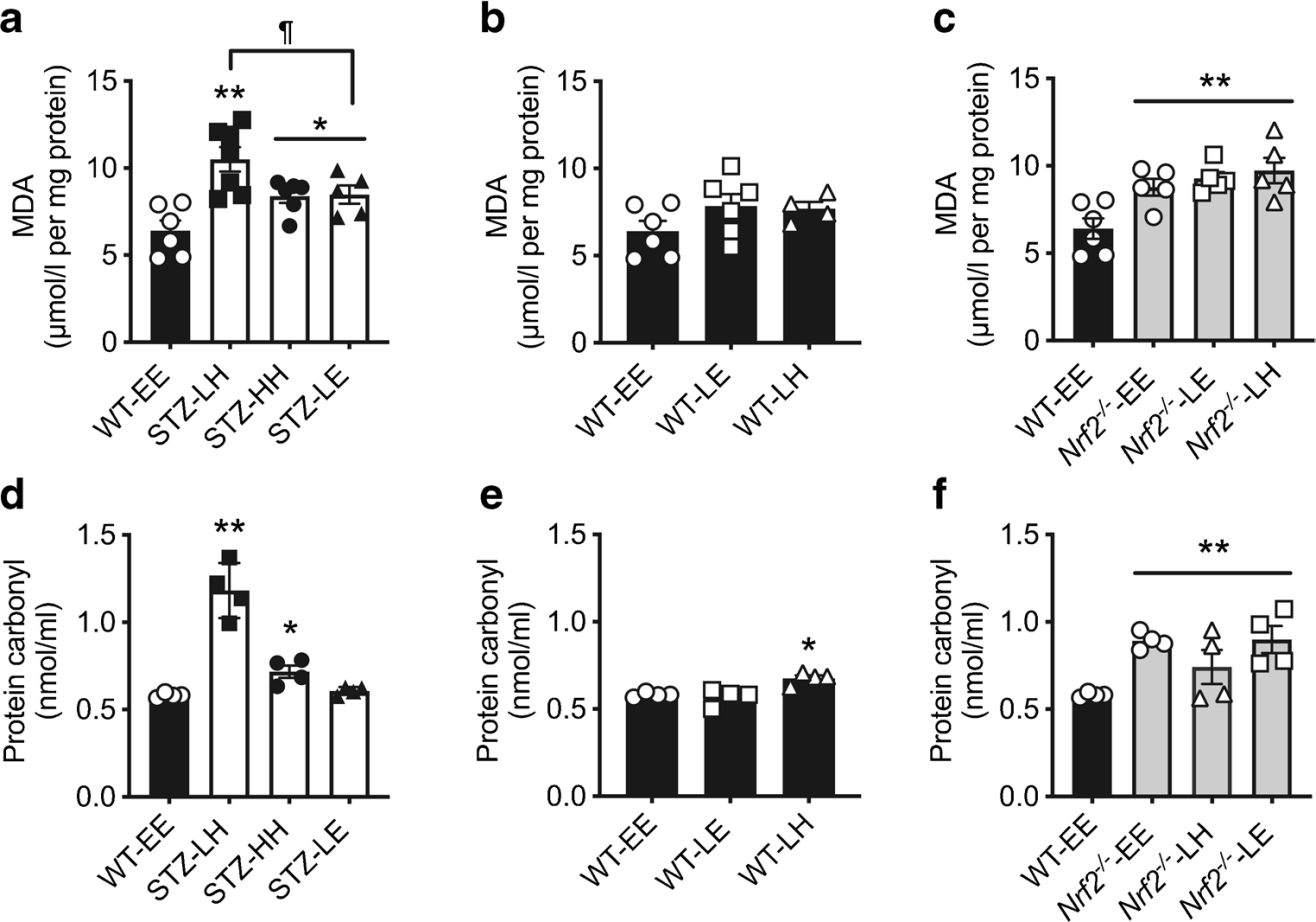
Chronic hyperglycaemia is associated with hippocampal oxidative damage. (**a**) Levels of hippocampal lipid peroxidation were increased in STZ-diabetic mice (white bars) compared with control (WT) mice (black bars) maintained at euglycaemia. (**b**) Euglycaemic control mice exposed to an acute episode of hypoglycaemia exhibited no change in hippocampal lipid peroxidation. (**c**) Euglycaemic *Nrf2*^−/−^ mice (grey bars) displayed increased levels of hippocampal lipid peroxidation irrespective of hypoglycaemic challenge. (**d**) Protein carbonylation levels were elevated in STZ-diabetic mice exposed to hyperglycaemia compared with control mice at euglycaemia. (**e**) Control WT mice exposed to an acute hypoglycaemic episode showed a rise in protein carbonylation only when recovered to a hyperglycaemic state. (**f**) *Nrf2*^−/−^ mice displayed increased levels of protein carbonylation irrespective of glycaemic variability. *n*=4–7/group. Results represent mean values ± SEM. *p<0.05, **p<0.01 vs WT-EE; ^¶^*p*<0.05 vs STZ-diabetes (one-way ANOVA followed by Tukey post hoc test). E, euglycaemia; H, high, hyperglycaemia; L, low, hypoglycaemia; MDA, malondialdehyde

In non-diabetic WT control mice, acute hypoglycaemia did not significantly increase lipid peroxidation irrespective of the glucose level at which the clamp finished (Fig. 2b; WT-EE vs WT-LE, *p*>*0.05*; WT-EE vs WT-LH, *p*>0.05). In contrast, hippocampal lipid peroxidation was significantly increased in all STZ-diabetic models, with the most significant effect seen where there was post-hypoglycaemic hyperglycaemia (Fig. 2a; STZ-LH vs WT-EE, *p*<0.01). In STZ-diabetes, maintaining post-hypoglycaemic euglycaemia ameliorated this effect (STZ-LE vs STZ-LH, *p*<0.05). The levels of lipid peroxidation in *Nrf2*^−/−^ mice were elevated in all conditions when compared with control (WT-EE) mice (Fig. 2c; main effect of genotype, *p*<0.01).

Protein carbonylation is commonly used as a biomarker of oxidative damage for many proteins. Levels increase with age, and this increase has been linked to changes in specific enzymes, such as members of the tyrosine kinase family [30], GLUT4 [31] and the 19s and 20s proteasomal subunits [32], and to diseases such as diabetes [33–35]. In non-diabetic WT mice, there was no impact of a single acute hypoglycaemic challenge on levels of carbonylated proteins when returned to euglycaemic levels (Fig. 2e; WT-EE vs WT-LE, *p*>0.05). In contrast, in STZ-diabetic mice, hypoglycaemia followed by recovery to hyperglycaemia resulted in a marked increase in protein carbonylation (Fig. 2d; WT-EE vs STZ-LH, *p*<0.01). There were also small but significant increases in carbonylated protein levels in STZ-diabetic mice that had not been exposed to hypoglycaemia (Fig. 2d; WT-EE vs STZ-HH, *p*<0.05), as well as non-diabetic mice who were exposed to post-hypoglycaemic hyperglycaemia (Fig. 2e; WT-LH vs WT-EE, *p*<0.05). Interestingly, recovery of STZ-diabetic mice to euglycaemia largely reversed the increase in protein carbonylation (Fig. 2d; WT-EE vs STZ-LE, *p*>0.05). Notably, levels of carbonylated proteins were significantly elevated in the hippocampus of all *Nrf2*^−/−^ mice compared with non-diabetic WT mice (Fig. 2f; main effect of genotype, *p*<0.01).

### SILAC quantitative proteomics reveals changes in markers of cellular stress responses to hypoglycaemia

SILAC is a method of accurately quantifying changes in protein expression [24]. In vivo SILAC with label-free proteomics was used to assess changes in hippocampal protein expression in STZ-diabetic mice exposed to post-hypoglycaemic hyperglycaemia (STZ-LH, group E) compared with control mice (WT-EE, group A). This procedure identified 71 proteins that were differentially expressed between groups (ESM Table 3 [upregulated proteins] and ESM Table 4 [downregulated proteins]). Pathway analysis identified significant upregulation of proteins involved in long-chain fatty acid metabolism (predominantly β-oxidation) and components of the proteasome, suggesting an enhanced capacity for long-chain fatty acid oxidation and the degradation of damaged proteins (ESM Table 3). Conversely, significant downregulation of proteins involved in mediating the stress response, including several heat shock proteins, was observed (ESM Table 4).

### Dysfunction of markers of protein chaperone function following hypoglycaemia in diabetes

**Fig. 3:**
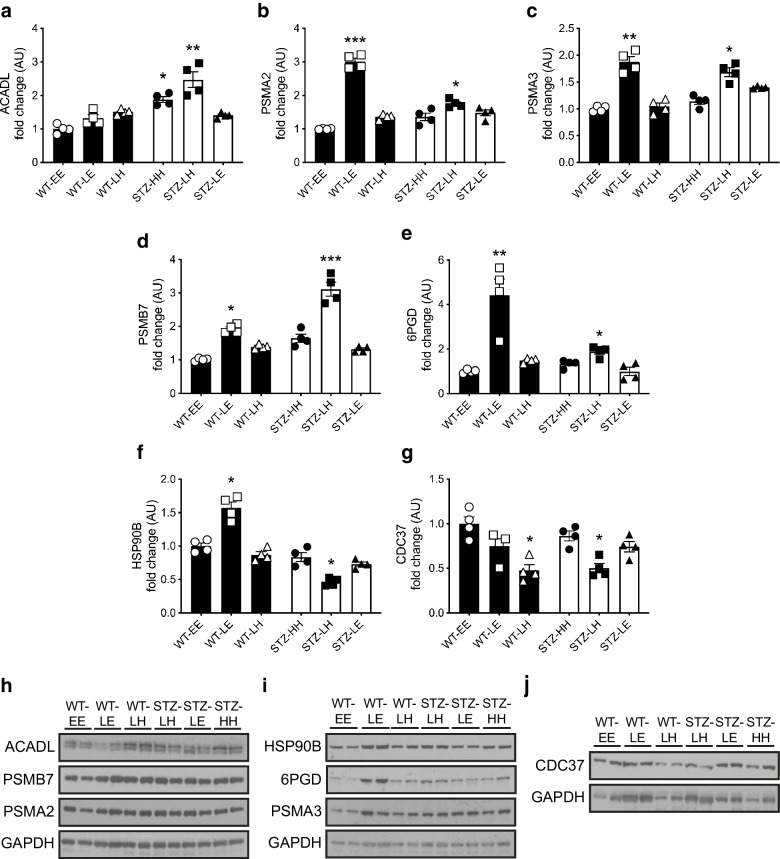
Effect of glycaemic variability on SILAC-outcome selected protein levels in the hippocampus of WT control and STZ-diabetic mice. (**a**–**g**) Hippocampal protein levels (ratio of signal intensities to control euglycaemia [WT-EE] data) in mice exposed to an acute hypoglycaemic episode from a euglycaemic (WT control mice, black bars) or hyperglycaemic (STZ-diabetic mice, white bars) baseline and returned to euglycaemia or hyperglycaemia: ACADL (**a**); PSMA2 (**b**); PSMA3 (**c**); PSMB7 (**d**); 6PGD (**e**); HSP90B (**f**); and CDC37 (**g**). (**h**–**j**) Representative immunoblots of ACADL, PSMB7 and PSMA2 (**h**), HSP90B, 6PGD and PSMA3 (**i**), and CDC37 (**j**) and their respective GAPDH loading controls. Results represent mean values ± SEM. *p<0.05, **p<0.01, ***p<0.001 (Kruskal–Wallis one-way ANOVA followed by Dunn’s multiple comparisons test). E, euglycaemia; H, high, hyperglycaemia; L, low, hypoglycaemia

We then examined candidate proteins from the key pathways identified in the SILAC analysis (fatty acid metabolism, proteasomal degradation and chaperone/stress response) across all study groups. ACADL, a mitochondrial protein involved in the initial step of fatty acid β-oxidation, was increased following hypoglycaemia in STZ-diabetic mice, an effect that was not seen when glucose was recovered to euglycaemia (Fig. 3a). In addition, we considered upregulation of 6PGD of interest in relation to the oxidative damage associated with the post-hypoglycaemic hyperglycaemic phase (ESM Table 3). 6PGD is a key enzyme of the oxidative arm of the pentose phosphate pathway (PPP) and the largest contributor to cytosolic NADPH, an important component of cellular antioxidant defences. 6PGD was enhanced in control and STZ-diabetic mice exposed to an acute hypoglycaemic challenge compared with control mice, although the impact of hypoglycaemia was less pronounced in STZ-diabetic mice (Fig. 3e, i; WT-EE vs STZ-LH, *p*<0.05; WT-EE vs WT-LE, *p*<0.01).

PSMA2 (Fig. 3b, h), PSMA3 (Fig. 3c, i) and PSMB7 (Fig. 3d, h), which form part of the 20S core structure, were all significantly increased following exposure to hypoglycaemia in both non-diabetic and STZ-diabetic mice (all *p*<0.05). HSP90, a chaperone protein that assists in correct protein folding and aids degradation of damaged proteins [36], was reduced following hypoglycaemia in STZ-diabetic mice (Fig. 3f, i; WT-EE vs STZ-LH, *p*<0.05). This contrasts with non-diabetic mice where acute hypoglycaemic challenge induced an increase in expression of HSP90B (Fig. 3f, i; WT-EE vs WT-LE, *p*<0.05). Similarly, hypoglycaemia in STZ-diabetic but not non-diabetic mice downregulated CDC37, an HSP90B co-chaperone protein (Fig. 3g). Interestingly, this effect was lost when STZ-diabetic mice were recovered to euglycaemia; however, CDC37 was also suppressed in non-diabetic mice recovered from hypoglycaemia to hyperglycaemia, suggesting that post-hypoglycaemic hyperglycaemia suppresses CDC37.

**Fig. 4:**
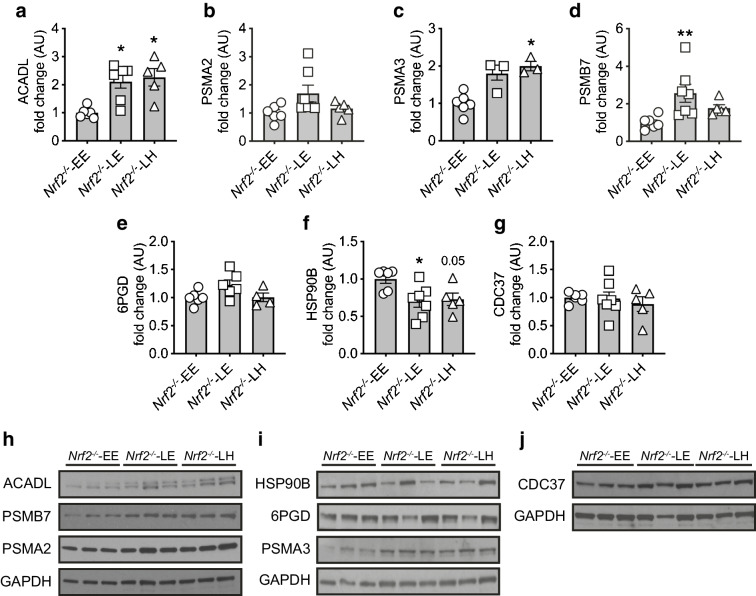
Effect of glycaemic variability on SILAC-outcome selected protein levels in the hippocampus of *Nrf2*^− /−^ mice. (**a**–**g**). Hippocampal protein levels (ratio of signal intensities to *Nrf2*^*-/-*^ mice at euglycaemia [*Nrf2*^*-/-*^*-*EE] data) in *Nrf2*^−/−^ mice exposed to an acute hypoglycaemic episode from a euglycaemic baseline and returned to euglycaemia or hyperglycaemia: ACADL (**a**); PSMA2 (**b**); PSMA3 (**c**); PSMB7 (**d**); 6PGD (**e**); HSP90B (**f**); and CDC37 (**g**). (**h**–**j**) Representative immunoblots of ACADL, PSMB7 and PSMA2 (**h**), HSP90B, 6PGD and PSMA3 (**i**), and CDC37 (**j**), and their respective GAPDH loading controls. Results represent mean values ± SEM. *p<0.05, **p<0.01 (Kruskal–Wallis one-way ANOVA followed by Dunn’s multiple comparisons test). E, euglycaemia; H, high, hyperglycaemia; L, low, hypoglycaemia

To further examine the role of NRF2 in mediating protection against the oxidative stress associated with both hyper- and hypoglycaemia, we also assessed the impact of acute changes in glycaemia on hippocampal levels of these proteins. Protein abundance of the mitochondrial protein ACADL was significantly elevated, whereas 6PGD did not increase in *Nrf2*^−/−^ mice (Fig. 4a, e). The increase in 6PGD was also seen in STZ-diabetic mice that had been exposed to acute hypoglycaemia euglycaemia (Fig. 3e). Exposure to hypoglycaemia increased the expression of PSMA3 and PSMB7 (Fig. 4c, i; *p*<0.05 vs WT-EE; and Fig. 4d, h; *p*<0.01 vs WT-EE) in *Nrf2*^−/−^ mouse hippocampus, with a non-statistically significant increase in PSMA2 (Fig. 4b, h; *p*=0.07). Similarly, the pattern of change in HSP90B after hypoglycaemia in *Nrf2*^−/−^ mice was also seen in STZ-diabetic mice but not non-diabetic WT mice who experienced post-hypoglycaemia (Fig. 4f). This suggests roles for NRF2 particularly in mediating the increase in reductive biosynthesis and chaperone/stress responses, which appear key pathways in the cellular response to hypoglycaemia.

## Discussion

In the present study, the hyperinsulinaemic clamp technique was combined with tissue analysis using SILAC proteomics and measures of oxidative stress to reveal a profound disruption in the cellular response to a hypoglycaemic challenge in a mouse model of chemically induced type 1 diabetes that increases the vulnerability of the hippocampus to oxidative damage. Of note, post-hypoglycaemic hyperglycaemia in STZ-diabetes was associated with a downregulation of proteins mediating the stress response and reductive biosynthesis. This is likely to result in proteotoxic stress through a reduced ability of cells to maintain the correct folding of proteins damaged by the stress challenge. This may, in turn, lead to irreversible damage/modification to proteins or synapses between cells within crucial brain regions such as the hippocampus.

In the current study, a single episode of hypoglycaemia in non-diabetic WT mice resulted in significant upregulation of *Nrf2* and NRF2 target genes *Hmox-1* and *Sod2* in WT control mice but with no oxidative damage. This supports long-term studies showing that recurrent non-severe hypoglycaemia in rodents without diabetes has no cognitive sequelae and may even be neuroprotective [13, 36]. NRF2 controls cellular adaptation to oxidative stress and increases during redox perturbation, inflammation and nutrient/energy fluxes, thereby enabling the factor to orchestrate adaptive responses to diverse forms of stress (for review, see [37]). The present study suggests that with a normally functioning NRF2-mediated response to cellular stress, there are no long-term cognitive sequelae to acute hypoglycaemia.

In contrast, when glucose levels were recovered from hypoglycaemia to hyperglycaemia, there was evidence of a small but significant increase in oxidative damage. This is consistent with prior work in neuronal cultures and in vivo models showing that hyperglycaemia after hypoglycaemia results in increased superoxide production and neuronal death [18]. It is of interest that the percentage increases in hippocampal lipid peroxidation and protein carbonylation in the present study is similar to those reported in transgenic mouse models [38, 39] and human post-mortem studies of Alzheimer’s disease [40, 41]. However, it is important to recognise that these represent chronic rather than acute disease models. Another notable finding in the present study is that the increase in hippocampal protein carbonylation was accompanied by a much smaller (1.67-fold vs 4.83-fold) increase in *Sod2* expression and the absence of *Hmox-1* upregulation in STZ-diabetes compared with non-diabetic WT mice exposed to post-hypoglycaemic hyperglycaemia. This indicates that chronic hyperglycaemia in diabetes may impair the ability to mount a robust antioxidant response.

SILAC labelling and quantitative proteomics of hippocampal tissue revealed that post-hypoglycaemic hyperglycaemia in STZ-diabetic mice resulted in an increased expression of several mitochondrial proteins involved in long-chain lipid-oxidation (hydroxyacyl-CoA dehydrogenase trifunctional multienzyme complex subunit α and β [HADHA and HADHB]), lipid transfer (sterol carrier protein 2 [SCP2]) and β-oxidation (ACADL). Previous research has shown a shift towards alternative fuel use following hypoglycaemia [42, 43]. Consistent with this, hypoglycaemia increased levels of ACADL in almost all groups in the current study, including *Nrf2*^−/−^ mice. The higher levels of ACADL seen in STZ-diabetic mice per se likely reflect increased lipid transport and β-oxidation because of chronic uncontrolled diabetes.

In contrast, clearer differences emerged between groups in the expression of a key enzyme, 6PGD, which sits within the oxidative arm of the PPP. The increase in 6PGD expression, while significant in STZ-diabetic mice exposed to hypoglycaemia, was much smaller than that induced in the non-diabetic WT control mice. Increased flux through this pathway increases the production of the reducing equivalent NADPH required for the reactive biosynthesis of fatty acids and cholesterol and the production of intermediates used in synthesising nucleotides. Increased levels of NADPH are also essential for ameliorating oxidative stress by reducing oxidised glutathione (GSH). Notably, there was no change in 6PGD expression in *Nrf2* null mice following hypoglycaemia. This finding is in keeping with a recent report demonstrating that NRF2 regulates the transcription of 6PGD through direct binding to the antioxidant response element within its promoter region [44]. Interruption of glucose supply with reduced PPP and NADPH generation, such as during a hypoglycaemic event in type 1 diabetes (where induction of *Sod2* and *Hmox-1* is impaired), will further hamper detoxification of ROS and the induction of antioxidant defence proteins. Indeed, previous work has shown that glucose withdrawal abrogates the induction of *Hmox-1* by the classical NRF2 activator sulforaphane [45]. This suggests that in STZ-diabetes, there is an impairment in reductive biosynthesis that may increase cellular vulnerability to oxidative stress.

Hypoglycaemia also increased the expression of proteasomal subunits (PSMA2, PSMA3 and PSMB7) in both control and STZ-diabetic mice. The proteasome is an integral part of the ubiquitin–proteasome system (UPS) and corresponding cellular protein quality control (PQC) [46]. If proteasome complex assembly and function are impaired, this can lead to reduced proteolytic activities and the accumulation of damaged or misfolded protein species [47]. In the present study, hypoglycaemia increased levels of proteasomal proteins in all groups, suggesting this response to an oxidative insult is intact, although the rise was less pronounced in *Nrf2*^−/−^ mice. NRF2 activation has been demonstrated to increase the expression of proteasomal genes and enhance the removal of oxidised proteins following oxidative insult, so this may contribute at least in part to the cellular response to hypoglycaemia [48, 49].

In contrast to the broadly similar impact of hypoglycaemia on the proteasome in all study groups, we found divergent effects of hypoglycaemia on the stress response protein HSP90B when comparing mice with and without STZ-diabetes. Other stress response proteins (heat shock protein 90, α [cytosolic], class A member 1 [HSP-90AA1], heat shock protein family H [HSP110] member 1 [HSPH1] and stress-induced phosphoprotein 1 [STIP1]) were also shown by SILAC to be downregulated in STZ-diabetic mice exposed to hypoglycaemia and recovered to hyperglycaemia. In addition, the HSP90B co-chaperone protein CDC37 was downregulated following acute hypoglycaemia in the STZ-diabetic mice. Interestingly, hypoglycaemia also decreased HSP90B in *Nrf2* null mice, independently from CDC37, indicating a possible involvement of NRF2 in this cell protective mechanism. Indeed, STIP1 plays an essential role in the ability of HSP90 to stabilise the NRF2–kelch-like ECH-associated protein 1 (KEAP1) complex [50], supporting functional connectivity between these important cellular stress response pathways. These data suggest that activation of stress response proteins is impaired in STZ-diabetic mice exposed to hypoglycaemia, leading to proteotoxic stress. Furthermore, NRF2 may be required for this aspect of the cellular response to hypoglycaemia.

Limitations of this study include the use of a chemically induced mouse model of type 1 diabetes that does not entirely replicate the human condition, the inclusion of only male mice, and the analysis being performed on the whole hippocampus rather than on isolated neurons or astrocytes. Additionally, lipid peroxidation and protein carbonylation measures provide a global oxidative damage index. Still, they do not allow the identification of specific proteins or pathways that may be directly impacted in this context. It would have been interesting to determine whether there was a correlation between the amount of oxidative damage, depth of hypoglycaemia and degree of post-hypoglycaemic hyperglycaemia, as demonstrated in neuronal cell cultures [18]. However, this requires multiple groups and is best studied ex vivo or in vitro. In addition, it would have been interesting to examine whether normalising glucose levels in the rodent type 1 diabetes model reversed the changes seen. Future studies are planned to address this question.

In conclusion, results from the present study suggest that a functioning NRF2-mediated response to cellular stress in non-diabetic rodents protects the hippocampus from any consequences due to acute non-severe hypoglycaemia. In contrast, in a mouse model of chemically induced type 1 diabetes, the chronic exposure to hyperglycaemia that characterises diabetes (especially when sub-optimally controlled) and post-hypoglycaemic hyperglycaemia result in sufficient oxidative stress to induce oxidative damage in the hippocampus and may then contribute to longer-term cognitive sequelae. Proteomic analysis of hippocampal tissue revealed evidence of disruption in proteins mediating the stress response and reductive biosynthesis in STZ-diabetes mice exposed to a single episode of non-severe hypoglycaemia. This is likely to result in proteotoxic stress through a reduced ability of cells to maintain the correct folding of proteins damaged by the stress challenge and may lead to irreversible damage modification to proteins or synapses between cells within crucial brain regions such as the hippocampus. Future research that more specifically examines underlying mechanisms in neurons, astrocytes and microglia may enable more targeted therapies, such as enhancing NRF2 activity. It is also important to consider the impact of reducing glycaemic variability prior to and/or following hypoglycaemia on oxidative stress in different brain regions.

### Supplementary information

ESM (PDF 358 kb)

## Data Availability

The datasets generated during and/or analysed during the current study are available in ProteomeXchange, accession no. 1-20220824-173727 (www.proteomexchange.org). Additional datasets generated during and/or analysed during the present study are available from the corresponding author upon reasonable request.

## Abbreviations

ACADL: Long-chain acyl Co-A dehydrogenase
CDC37: HSP90 co-chaperone
EDIC: Epidemiology of Diabetes Interventions and Complications
HSP90B: Heat shock protein 90-β
NRF2: Nuclear factor erythroid 2-related factor 2
6PGD: 6-Phosphogluconate dehydrogenase
PPP: Pentose phosphate pathway
PSMA2: Proteasome subunit α type-2
PSMA3: Proteasome subunit α type-3
PSMB7: Proteasome subunit β type-7
ROS: Reactive oxygen species
SILAC: Stable isotope labelling by amino acids in cell culture
STZ: Streptozotocin
WT: Wild-type
