# Correction: Chronic hyperglycaemia increases the vulnerability of the hippocampus to oxidative damage induced during post-hypoglycaemic hyperglycaemia in a mouse model of chemically induced type 1 diabetes

**DOI:** 10.1007/s00125-024-06188-3

**Published:** 2024-06-12

**Authors:** Alison D. McNeilly, Jennifer R. Gallagher, Mark L. Evans, Bastiaan E. de Galan, Ulrik Pedersen-Bjergaard, Bernard Thorens, Albena T. Dinkova-Kostova, Jeffrey-T. Huang, Michael L. J. Ashford, Rory J. McCrimmon, on behalf of the Hypo-RESOLVE Consortium

**Affiliations:** 1 Division of Systems Medicine, School of Medicine, Ninewells Hospital and Medical School, Dundee, UK; 2 Wellcome-MRC Institute of Metabolic Science, University of Cambridge, Cambridge, UK; 3 Radboud University Medical Center, Nijmegen, the Netherlands; 4 Department of Internal Medicine, Maastricht University Medical Center, Maastricht, the Netherlands; 5 CARIM School for Cardiovascular Diseases, Maastricht University, Maastricht, the Netherlands; 6 Nordsjællands Hospital Hillerød, University of Copenhagen, Hillerød, Denmark; 7 Faculty of Biology and Medicine, University of Lausanne, Lausanne, Switzerland; 8 Division of Cancer Research, School of Medicine, Ninewells Hospital and Medical School, Dundee, UK; 9 Biomarker and Drug Analysis Core Facility, School of Medicine, Ninewells Hospital and Medical School, Dundee, UK

**Correction: Diabetologia (2023) 66:1340-1352**

10.1007/s00125-023-05907-6

Unfortunately, the funding statement included in this paper did not fully satisfy the reporting requirements of the funder. The original article has been updated to include the sentence: ‘This paper reflects the authors’ views and the JU is not responsible for any use that may be made of the information it contains’.

