# Supplementary material for: Chronic hyperglycaemia increases the vulnerability of the hippocampus to oxidative damage induced during post-hypoglycaemic hyperglycaemia in a mouse model of chemically induced type 1 diabetes
Source: Diabetologia. 2023 Apr 4;66(7):1340–52. doi: 10.1007/s00125-023-05907-6 (PMC10244284; doi:10.1007/s00125-023-05907-6)
Supplement: Supplementary file 1 — (PDF 358 kb) [file 125_2023_5907_MOESM1_ESM.pdf]

ESM Table 1. Transcript abundance of Nrf2 target genes in Control and STZ- diabetic mice

	A (WT-EE)	B (WT-LE)	C (WT-LH)	D (STZ-HH)	E (STZ-LE)	F (STZ-LH)
Nrf2	1.0±0.04	0.98±0.05	1.16±0.12	1.48±0.03*	1.28±0.08*	1.79±0.12*
Nqo1	1.0±0.03	0.94±0.03	0.87±0.03	1.53±0.15*	1.39±0.19	1.53±0.09*
Sod2	0.99±0.04	4.83±0.03*	0.59±0.51	5.73±0.61*	0.69±0.06	1.67±0.11*
Hmox-1	0.99±0.09	1.42±0.10*	0.92±0.10	0.85±0.05	0.89±0.04	1.11±0.11

Control (WT) mice, groups A-C; Type 1 diabetic (STZ) mice, groups D-F. Clamp protocols: E = Euglycemia, L = Hypoglycaemia, H = Hyperglycaemia. Ratios presented relative (to A (EEE)) gene expression of NAD(P)H: quinoneoxidoreductase 1 (*Nqo1*), superoxide dismutase 2 (*Sod2*), hemeoxygenase 1 (*Hmox-1*), n = 10-12/group. Data were analysed by Kruskall-Wallis with Dunn's multiple comparisons test. Results represent mean values ± SEM. *$P < 0.05$, vs WT-EE

ESM Table 2. Transcript abundance of Nrf2 target genes in *Nrf2*$^{-/-}$ mice

	G (Nrf-EE)	H (Nrf2-LE)	I (Nrf2-LH)
Nqo1	1.0±0.04	0.99±0.09	0.94±0.03
Sod2	0.99±0.14	1.14±0.21	0.91±0.10
Hmox-1	0.99±0.18	1.18±0.32	0.97±0.10

Nrf2$^{-/-}$ mice, groups G-I. n = 8-10/group. Data were analysed by Kruskall-Wallis with Dunn's multiple comparisons test. Results represent mean values ± SEM

ESM Table 3. Upregulated proteins

Protein names	Gene names	Fold change	p value
Coiled-coil domain-containing protein 47	*Ccdc47*	1.28	0.0066
Non-specific lipid-transfer protein	*Scp2*	1.26	0.0279
Serine/threonine-protein phosphatase 4 regulatory subunit 2	*Ppp4r2*	1.24	0.0134
Alanine--tRNA ligase, cytoplasmic	*Aars*	1.18	0.0050
Thyroid hormone receptor-associated protein 3	*Thrap3*	1.18	0.0075
SWI/SNF complex subunit SMARCC2	*Smarcc2*	1.18	0.0434
Trifunctional enzyme subunit alpha, mitochondrial;Long-chain enoyl-CoA hydratase;Long chain 3-hydroxyacyl-CoA dehydrogenase	*Hadha*	1.14	0.0233
Phosphatidylinositol 5-phosphate 4-kinase type-2 gamma	*Pip4k2c*	1.13	0.0100
Trifunctional enzyme subunit beta, mitochondrial;3-ketoacyl-CoA thiolase	*Hadhb*	1.13	0.0242
Proteasome subunit alpha type-2	*Psma2*	1.13	0.0439
Small acidic protein	*Smap;1110004F10Rik*	1.12	0.0396
6-phosphogluconate dehydrogenase, decarboxylating	*6Pgd*	1.11	0.0195
Long-chain specific acyl-CoA dehydrogenase, mitochondrial	*Acadl*	1.11	0.0369
Lupus La protein homolog	*Ssb*	1.10	0.0244
Isoleucine--tRNA ligase, mitochondrial	*Iars2*	1.09	0.0130
D-beta-hydroxybutyrate dehydrogenase, mitochondrial	*Bdh1*	1.09	0.0446
Ornithine aminotransferase, mitochondrial	*Oat*	1.09	0.0426
Heterochromatin protein 1-binding protein 3	*Hp1bp3*	1.09	0.0222
Carbonyl reductase [NADPH] 1	*Cbr1*	1.09	0.0023
Plastin-3	*Pls3*	1.09	0.0312
Proteasome subunit alpha type-3	*Psma3*	1.09	0.0186
Neurochondrin	*Ncdn*	1.09	0.0342
Fructose-bisphosphate aldolase C	*Aldoc*	1.08	0.0289
Lamin-B1	*Lmnb1*	1.08	0.0180
60S acidic ribosomal protein P1	*Rplp1;Gm10073*	1.08	0.0384
Succinyl-CoA:3-ketoacid coenzyme A transferase 1, mitochondrial;Succinyl-CoA:3-ketoacid-coenzyme A transferase	*Oxct1*	1.07	0.0196
Proteasome subunit beta type-7	*Psmb7*	1.07	0.0403
Electron transfer flavoprotein subunit alpha, mitochondrial	*Etfa*	1.06	0.0343
Nucleolin	*Ncl*	1.06	0.0493
NADH dehydrogenase [ubiquinone] 1 beta subcomplex subunit 3	*Ndufb3*	1.06	0.0388
Protein DJ-1	*Park7*	1.05	0.0162
Ubiquitin carboxyl-terminal hydrolase;Ubiquitin carboxyl-terminal hydrolase 14	*Usp14*	1.05	0.0228
Acyl carrier protein;Acyl carrier protein, mitochondrial	*Ndufab1*	1.05	0.0185
NADH dehydrogenase [ubiquinone] iron-sulfur protein 6, mitochondrial	*Ndufs6*	1.05	0.0401
Aldehyde dehydrogenase, mitochondrial	*Aldh2*	1.05	0.0137
Phosphoglucomutase-1	*Pgm1;Pgm2*	1.05	0.0285
Actin-binding LIM protein 1	*Ablim1*	1.04	0.0459
Guanine nucleotide-binding protein G(i) subunit alpha-1	*Gnai1*	1.03	0.0014

ESM Table 4. Downregulated proteins

Protein names	Gene names	Fold change	p value
Ubiquitin carboxyl-terminal hydrolase;Ubiquitin carboxyl-terminal hydrolase 5	*Usp5*	0.96	0.0164
Myosin-10	*Myh10*	0.96	0.0469
Microtubule-associated protein tau	*Mapt*	0.96	0.0397
AP2-associated protein kinase 1	*Aak1*	0.95	0.0331
Heat shock cognate 71 kDa protein	Hspa8	0.95	0.0234
Epidermal growth factor receptor substrate 15-like 1	*Eps15l1*	0.95	0.0390
Thy-1 membrane glycoprotein	*Thy1*	0.95	0.0401
Calcineurin subunit B type 1	*Ppp3r1*	0.95	0.0228
Cytosolic acyl coenzyme A thioester hydrolase	*Acot7*	0.95	0.0341
Kinesin-like protein KIF2A	*Kif2a*	0.94	0.0219
AP-3 complex subunit delta-1	*Ap3d1*	0.94	0.0189
Synaptosomal-associated protein 25	*Snap25*	0.93	0.0166
Glutaredoxin-3	*Glrx3*	0.93	0.0139
Serine/threonine-protein kinase PAK 1	*Pak1*	0.93	0.0100
Elongation factor Tu, mitochondrial	*Tufm*	0.93	0.0016
Cysteine and histidine-rich domain-containing protein 1	*Chordc1*	0.92	0.0410
Gamma-aminobutyric acid receptor-associated protein-like 2	*Gabarapl2*	0.91	0.0118
Cofilin-2	*Cfl2*	0.91	0.0428
Coronin;Coronin-1A	*Coro1a*	0.91	0.0407
Stress-induced-phosphoprotein 1	*Stip1*	0.90	0.0003
Rho-associated protein kinase 2	*Rock2*	0.90	0.0451
AMP deaminase 2	*Ampd2*	0.90	0.0109
Mitochondrial glutamate carrier 1	*Slc25a22*	0.90	0.0176
Calcyclin-binding protein	*Cacybp*	0.89	0.0300
Glycogen debranching enzyme	*Agl*	0.88	0.0397
Alpha-adducin	*Add1*	0.88	0.0323
Hsp90 co-chaperone Cdc37	*Cdc37*	0.88	0.0101
Heat shock protein HSP 90-alpha	*Hsp90aa1*	0.88	0.0119
Peptidyl-prolyl cis-trans isomerase D	*Ppid*	0.88	0.0089
Heat shock protein HSP 90-beta	*Hsp90ab1*	0.87	0.0261
Glucose 1,6-bisphosphate synthase	*Pgm2L1*	0.87	0.0226
Protein lunapark	*Lnp*	0.86	0.0127
ETS translocation variant 2	*Etv2*	0.86	0.0439
Clusterin;Clusterin beta chain;Clusterin alpha chain	*Clu*	0.86	0.0078
cGMP-dependent 3,5-cyclic phosphodiesterase	*Pde2a*	0.85	0.0394
Nuclear migration protein nudC	*Nudc*	0.85	0.0221
Small glutamine-rich tetratricopeptide repeat-containing protein beta	*Sgtb*	0.83	0.0130
Heat shock protein 105 kDa	*Hsph1*	0.83	0.0028
CD99 antigen-like protein 2	*Cd99l2*	0.78	0.0324
Hydroxymethylglutaryl-CoA synthase, cytoplasmic	*Hmgcs1*	0.78	0.0388
5-nucleotidase domain-containing protein 3	*Nt5dc3*	0.72	0.0267
